# Surgical site infections by atypical mycobacteria: prevalence and species characterization using MALDI-TOF and molecular LCD chip array

**DOI:** 10.1007/s15010-022-01864-1

**Published:** 2022-06-18

**Authors:** Maha A. Gad, Sahar M. Khairat, Amira M. A. Salama, Omnia A. Abd Elmoez, Noha S. Soliman

**Affiliations:** 1 Department of Clinical and Chemical Pathology, Faculty of Medicine, Cairo University, Cairo, Egypt; 2 Department of Clinical and Chemical Pathology, Faculty of Medicine, Cairo University, Cairo, Egypt; 3 Central Public Health Laboratories (CPHL), Ministry of Health, Cairo, Egypt

**Keywords:** Surgical site infections, Non-tuberculous mycobacteria, MALDI-OF MS, LCD array, *M. abscessus*, *M. porcinum*

## Abstract

**Background:**

Surgical site infection (SSI) is a post-operative complication of high concern with adverse impact on patient prognosis and public health systems. Recently, SSI pathogens have experienced a change in microbial profile with increasing reports of non-tuberculous mycobacteria (NTM) as important pathogens.

**Aim:**

of the study

The study aimed to detect the prevalence of NTM among cases with SSIs and describe their species using matrix-assisted laser desorption ionization time of flight mass spectrometry (MALDI-TOF MS) and PCR-based microarray.

**Methods:**

The study was conducted with 192 pus samples collected from patients with SSI. Mycobacterial investigations were done in the form of Ziehl–Neelsen (ZN) smears for acid-fast bacilli, automated mycobacterial culture to isolate mycobacteria, followed by immunochromatography test to predict NTM. NTM-positive cultures were tested by MALDI -TOF MS and PCR-based microarray to reach species-level identification.

**Results:**

Mycobacterial growth was found in 11/192 samples (5.7%) and identified as 4 NTM and 7 M*. tuberculosis* isolates with prevalence of 2.1% and 3.64%, respectively. The NTM species were described by MALDI-TOF as *M. abscessus, M. porcinum, M. bacteremicum,* and *M. gordonae.* Microarray agreed with MALDI-TOF in identifying one isolate *(M. abscessus),* while two isolates were classified as belonging to broad groups and one isolate failed to be identified.

**Conclusions:**

The prevalence of NTM among SSI was found to be low, yet have to be considered in the diagnosis of mycobacteria. Employing advanced technologies in diagnosis is recommended to guide for appropriate treatment.

## Introduction

Surgical site infection (SSI) is one of the most common forms of post-operative complications and hospital-acquired infections [1, 2]. It adversely affects patients by causing delayed wound healing, increased length of hospital stay, prolonged use of antibiotics and increased mortality [3].

For proper management of infectious diseases, it is essential to accurately identify the causative pathogens. The surveillance data of the last two decades suggest a change in the profile of microorganisms causing SSIs, with available reports on the occurrence of SSIs by mycobacteria [4]. *M. tuberculosis* (MTB) attracts most global concern; however, infections caused by non-tuberculous mycobacteria (NTM) exist and may be overlooked due to limitations of conventional diagnostics in reaching accurate identification. Several reports on infections by NTM have been lately presented due to the achieved advance in diagnostic technologies [4–6]. Atypical NTM have existed in nature over the years and reported in history as causative agents for different types of infections including post-operative infections [7]. It is postulated that contaminated water or surgical instruments might be possible sources of post-operative infection [6]. It has been reported that rapid grower NTM are responsible for most of atypical mycobacterial cutaneous and soft tissue surgical infections, where environmental contamination is usually the source [8, 9]. Clinical diagnosis of mycobacterial infections is considered challenging, and suspicion is usually built on late, mild, however persistent clinical presentation with poor response to antibiotics [10, 11]. Missed diagnosis may lead to inappropriate treatment and unfavorable emergence of antimicrobial resistance; therefore, appropriate laboratory diagnosis is considered essential [12, 13]. Moreover, in mycobacterial infections, it is crucial to differentiate atypical mycobacteria from *M. tuberculosis* due to their different therapeutic regimens [5], which necessitates the provision of rapid and accurate laboratory diagnostic methods for proper identification [12, 13].

Traditionally, the identification of NTM was carried out by conventional phenotypic biochemical tests. However, these methods were laborious and unable to identify a wide spectrum of species [14]. In this context, many researchers employed the advanced developed technologies such as matrix-assisted laser desorption ionization–time of flight mass spectrometry (MALDI-TOF MS) and molecular DNA hybridization LCD chip array in identification of mycobacteria offering more rapid and accurate diagnosis [15, 16]. To this end, the present work aimed to study the prevalence of atypical mycobacteria among patients with SSI and characterize their species using MALDI-TOF assay and DNA hybridization LCD chip array.

## Materials and methods

### Study design

This study was conducted with a total of 192 non-repeat pus samples prospectively collected from patients evaluated at surgery outpatient clinics of Kasr Al-Ainy Hospital in Cairo University in the period from January 2020 to January 2021. The charged physician asked for mycobacterial culture for patients who developed symptoms and signs of post-operative SSI, with failed microbial isolation in usual bacterial culture and no improvement on prescribed antibiotics suggestive of mycobacterial etiology. Demographic data were collected from the electronic records on the laboratory information system for patients infected by NTM.

### Sample collection

Pus samples were collected from surgical sites through needle aspiration or a surgical procedure under complete aseptic precautions. Swabs were not recommended because of limited culture material. All samples were transported with no delay to the main mycobacteriology laboratory following the required biosafety measures. Full mycobacterial investigations were carried out in the form of ZN smears, automated culture for isolation of mycobacteria, followed by identification and differentiation between atypical mycobacteria and *M. tuberculosis* using immunochromatography assay, phenotypic MALDI-TOF assay and molecular LCD chip array.

### ZN smears

Smears were prepared from the thickest purulent part of the specimen. Dried and fixed smears were stained according to Ziehl–Neelsen procedure, then examined microscopically for the presence of acid-fast bacilli (AFB) [14].

### Isolation of mycobacteria using automated MGIT culture

Mycobacterial growth indicator tube (MGIT) culture was held using the BD BACTEC^™^ MGIT^™^ 960 system (Becton–Dickinson, Franklin Lakes, NJ, USA), which is a high-capacity continuous monitoring growth system. This automated system is reported to be faster and more reliable than the conventional culture on Lowenstein–Jensen (LJ) solid medium [14, 15]. The collected samples were inoculated in (MGIT) liquid culture method, with enrichment supplement (oleic acid–albumin–dextrose–citric acid [OADC]) and antimicrobial supplement (polymyxin B, nalidixic acid, trimethoprim, and azlocillin [PANTA]). Inoculated tubes were incubated in the device for up to a period of 40 days. Positive mycobacterial growth in MGIT culture was confirmed by making ZN smears to examine for the presence of AFB and secondarily inoculated on LJ medium to increase harvest for further identification.

### Identification of non-tuberculous *Mycobacterium* (NTM)

In positive MGIT growth cultures, the NTM isolates were differentiated from *M. tuberculosis* by their rapid growth (within 7 days incubation), colony morphology and pigment production on LJ media. NTM identification was confirmed by negative reaction with immune-chromatography assay (BD MGIT TBc ID, Beckton Dickinson Diagnostic, Sparks, USA). In this assay, 100 μl of the positive liquid culture was used to fill the sample well according to the manufacturer’s instructions. The principle of the test relied on a clearly pink to red visible line within 15 min that can be read when the antigen MPT64 (a mycobacterial protein secreted from *M. tuberculosis complex* cells) binds to the anti-MPT64 monoclonal antibody conjugated on the test strip [16, 17].

### Species characterization of NTM using MALDI-TOF and LCD array

The isolated NTM in MGIT culture were characterized to the species level with the use of MALDI-TOF Biotyper microflex LT system mass spectrometer (Bruker Daltonics, Bremen, Germany) and LCD Array Myco^Direct^ 1.7 (Chipron GmbH, Berlin, Germany). The steps of the identification process by MALDI-TOF MS involves inactivation and extraction; then, unique fingerprints for the extracted proteins were generated for analysis. Protein extraction was performed from solid *Mycobacterium* according to the manufacturer’s instructions and the protocol used in several studies [18, 19]. Following extraction, an aliquot was placed onto a steel plate and covered with a chemical matrix, then loaded on the device where protein ionization occurred by laser. The ions were separated according to the mass/charge (*m/z*) ratio of 2,000–20,000 Da. The generated proteomic profiles were analyzed through the database of Mycobacteria Library Software v2.46. Reliable identification is assessed based on the highest similarity between spectra from samples and in the database. According to the Bruker system, a score value of ≥ 1.8 codes for “high confidence genus and species identification”; however, a value of ≥ 1.6–1.79 denotes “low confidence species identification” [18].

Positive MGIT cultures were tested by low-cost, low-density (LCD) DNA microarray for NTM identification using LCD Array Myco^Direct^ 1.7 kit (CHIPRON GmbH, Berlin, Germany), which is a DNA hybridization kit designed for identification of *M. tuberculosis* complex and other non-tuberculous mycobacteria. It is based on two polymerase chain reactions which are combined prior to hybridization into one array. Identifying non-tuberculous *Mycobacterium* depends on Primer mix A, which is directed against highly conserved motifs flanking the internal transcribed spacer region (ITS) of the 16SrRNA gene, whereas *M. tuberculosis* is identified by primer mix B, which is directed against the insertion element *IS6110* [20]. All procedures were performed following the manufacturer’s instructions. The LCD chip was scanned using Chip Scanner PF 2700 and the data were analyzed by Slide Reader V7.00.01 (CHIPRON GmbH, Berlin, Germany).

### Statistical analysis

Data were coded, entered and analyzed using the Statistical Package for the Social Sciences (SPSS) version 28 (IBM Corp., Armonk, NY, USA). Data were described and calculated as frequencies (number of cases) and percentages. Sensitivity, specificity and 95% confidence interval were measured for ZN microscopy compared to the gold standard culture.

## Results

**Fig. 1:**
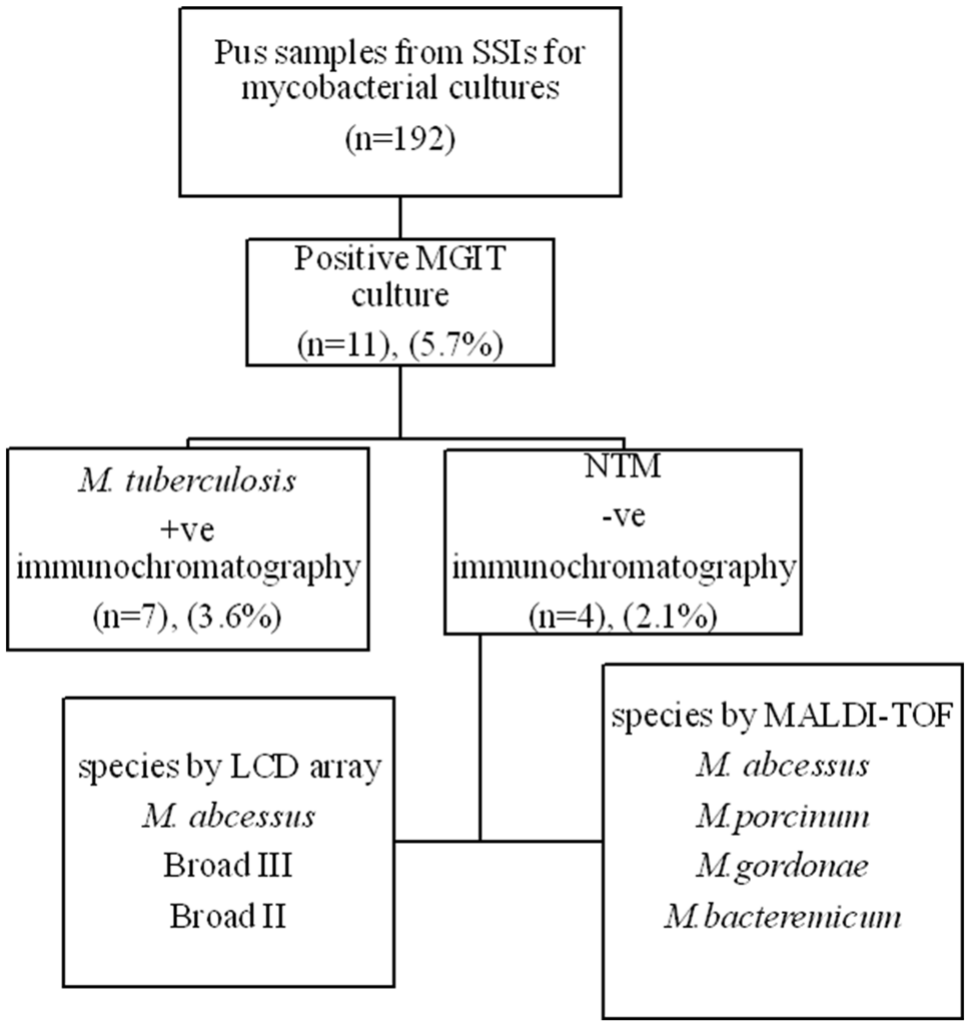
Flowchart of mycobacterial laboratory workshop results of pus samples (*n* = 192)

Out of a total 192 processed pus samples of patients suspected with mycobacterial infections by charged physicians, MGIT culture showed positive mycobacterial growth in 11 samples (5.7%). Compared to the gold standard mycobacterial culture, ZN smear showed true positive results in seven samples (smear positive/culture positive); however, four samples had false-negative smear results (smear negative/culture positive) and no samples showed false-positive smears. ZN smear showed a sensitivity of 63.6% (95% confidence interval: 0.316–0.87) and specificity of 100% (95% confidence interval: 0.97–1.00). The results of mycobacterial laboratory workup of pus samples (*n* = 192) are illustrated as shown in Fig. 1.

The phenotypic characteristics of the grown isolates and the immunochromatography assay divided the isolated mycobacteria into *M. tuberculosis* (*n* = 7) and NTM (*n* = 4), with prevalence of 3.64% and 2.1% of 192 suspected pus samples, respectively. NTM accounted for 36.4% of mycobacterial isolates: three were classified as rapid growers and one as a slow grower (Table 1).

**Table 1:** Growth characterization and species identification of NTM isolates with clinical data for infected patients (*n* = 4)

Isolate no	MALDI-TOF MS		LCD array	Growth characterization			Intervention	H/O
	Species	Score^**a**^		Rate of growth	Pigment	Colony morphology		
1.	*M. abscessus*	1.81	*M. abscessus*	Rapid growing	Non-pigmented	Smooth	Mesotherapy injection	DM
2.	*M. porcinum*	1.82	Broad IIIb	Rapid growing	Non-pigmented	Rough	Open laparotomy	Chemotherapy smoker
3.	*M. gordonae*	1.64	Broad II c	Slow growing	Scoto-chromogen	Smooth, yellow	Laparoscopic operation	Smoker
4.	*M. bacteremicum*	1.69	Unidentified	Rapid growing	Scoto-chromogen	Yellow	Open laparotomy	Smoker DMcancer

^a^MALDI-TOF score value for confidence level of identification; a) 1.80–3.00: high confidence and secure genus and species identification, b) 1.60–1.79: secure genus identification with low confidence species identification, c) 0.00–1.59: no reliable identification. DM: diabetes mellitus. H/O: patient history of risk factors

^b^ Broad III group of undifferentiated NTM including *M. porcinum*

^c^ Broad II group of undifferentiated NTM including *M. gordonae*

The species of the four NTM isolates were characterized by MALDI-TOF MS and LCD array as described in Table 1. Using MALDI-TOF MS, NTM isolates were identified as *M. abscessus* (high confidence), *M. porcinum* (high), *M. gordonae* (low), and *M. bacteremicum* (low). The LCD array was able to characterize the species of three out of four NTM isolates, but one isolate failed to be identified. One NTM species was identified as *M*. *abscessus* in agreement with MADI-TOF identification, while the two other NTM were broadly classified as one belonging to broad II and the other belonging to broad III. These isolates could not be identified to species level, as many mycobacterial species deliver PCR fragments of equal or similar size that evolved in a group known as “Broad”.

Patients infection by NTM (*n* = 4) had a history of open abdominal operations except one who had laparoscopic surgery and another who had mesotherapy injection. Three patients were male smokers and two of them were diabetic, while no one had a history of HIV infection (Table 1).

## Discussion

Atypical NTM is not of lesser concern than *M. tuberculosis* in causing post-operative infections [21]. To the best of our knowledge, data on the prevalence and types of NTM among SSI patients in our country are still limited. In an effort to bridge this gap, we tried to highlight the importance of considering atypical NTM among post-operative mycobacterial infections and avoid being mistaken for MTB. Moreover, there is a need to emphasize the role of employing MALDI-TOF and PCR-based array technologies in improving mycobacterial identification and encourage their adoption in developing countries according to the available financial resources.

In the present study, mycobacterial isolates were grown with prevalence of 3.64% and 2.1% for MTB and NTM, respectively. The NTM rate was consistent with that reported by a study in India (1.2%) [22]. Another Indian study reported higher NTM rates (10.9%), which likely occurred in another Brazilian study that confirmed NTM in 144 out 1051 suspected surgical mycobacterial infections [4, 21]. Variable rates might be due to different geographical areas, study population, types of samples and methods of detection. Of note, most of the previously published reports on NTM were among pulmonary or extra-pulmonary infections other than SSI and limited publications were available for NTM among SSI, apart from some studies on outbreaks, case reports or case series [6, 8]. In our study, NTM were isolated from four patients who underwent open operation, mesotherapy injection or laparoscopic procedure, mostly suggested to have originated from contaminated water or equipment [10, 23, 24].

In terms of laboratory diagnosis, ZN smear is considered a simple and cost-effective method, but of low-test sensitivity [25]. This was supported by ZN sensitivity of our study (63.6%) which well matched with that reported by another study (66.9%) [25]. In our study, we used immunochromatography BD MGIT^™^ assay that was encouraged by several studies for initial differentiation of NTM from *M. tuberculosis* [17, 26]. MALDI-TOF and LCD array were used for further characterization of the four recovered NTM isolates from pus culture. MALDI-TOF managed to identify four NTM species, where confident species identification was high in two isolates, however low in the other two isolates. This was as guided by the manufacturer; however, no global cutoff has been established for the species-level identification of the entire range of mycobacteria [18, 27–30]. In our study, the LCD array agreed with MALDI-TOF in characterizing one NTM isolate (*M. abscessus*); however, two NTM were broadly identified as Broad II and Broad III classes and one NTM was undetected. This could be explained by the many mycobacterial species delivering PCR fragments of similar size, thus evolving into a group known as “Broad” [31].

*M. abscessus* in our study was previously reported by several studies as a clinically important pathogen [10, 32]. *M. porcinum* was first described in pigs, while in human beings it was reported as a cause of cellulitis, central catheter infections, and pneumonitis [33, 34]. *M. bacteremicum* had previous reports of challenged diagnosis by VITEK MS, but was successfully detected by 16srRNA [35]. This species was previously reported from post-laparotomy infections [36]. *M. gordanae* was previously detected in post-traumatic as well as respiratory samples among immunosuppressed patients [37, 38].

In the last decade, few advanced technologies have evolved to overcome the flaws in conventional methods including being time consuming and only able to characterize a narrow spectrum of species [39, 40]. MALDI-TOF MS has been witnessed as a rapid and reliable tool for NTM identification, which includes a wide mycobacterial spectra in software databases, as many as 149 different *Mycobacterium* species [19]. However, some challenges were reported related to the extraction protocol, cost of the device and the need for a primary bacterial isolate [18, 19, 41–43]. LCD microarray is a rapid and reliable hybridization method with species-specific oligonucleotide probes that may reach a 100% sensitivity in mycobacterial identification [20, 44–47]. LCD microarray might not outperform MALDI-TOF due to a narrower spectrum of NTM species identification, besides its high cost; however, it has the privilege of enabling mycobacterial detection and identification directly from the sample, unlike MALDI-TOF [20, 31].

Of note, from our experience in work, the LCD array device is less expensive than MALDI-TOF as a diagnostic instrument; however, it has a higher running cost regarding testing a sample. After all, every healthcare setting shall evaluate the cost/benefit aspect in choosing the optimum diagnostic method to adopt in the laboratory and avoid underreporting due to defective diagnosis.

Our study had the following limitations: a) demographic data and type of surgery were available only for patients with NTM infections (*n* = 4); however, data for the rest of the patients were missing, b) LCD microarray could not be used for detection of mycobacteria directly from the samples due to financial barriers and was confined only to identifying the four recovered NTM isolates from pus culture.

## Conclusion

Clinicians should be aware of NTM in the diagnosis of mycobacterial surgical site infections. The present study detected four NTM isolates with a prevalence of 2.1% among surgical site infections submitted for mycobacterial culture. *M. abscessus, M. bacteremicum, M. porcinum, and M. gordanae* were characterized by MALDI-TOF. LCD array agreed with MALDI-TOF in identifying *M. abscessus*. Both assays play a vital role in improving mycobacterial identification, but require continuous update to expand the identification spectrum of mycobacterial species.

## Data Availability

All data generated or analyzed during this study are included in this published article.

## References

[1] Russo PL, Saguil E, Chakravarthy M (2021). Improving surgical site infection prevention in Asia-Pacific through appropriate surveillance programs: challenges and recommendation. Infect Dis Health.

[2] Behera HS, Chayani N, Bal M (2021). Identification of population of bacteria from culture negative surgical site infection patients using molecular tool. BMC Surg.

[3] Mehtar S, Wanyoro A, Ogunsola F (2020). Implementation of surgical site infection surveillance in low–and middle-income countries: a position statement for the international society for infectious diseases. Int J Infect Dis.

[4] Bhalla GS, Grover N, Singh G (2021). Prevalence of non-tuberculous mycobacterial infection in surgical site infections and their antibiotic susceptibility profile. Med J Armed Forces India.

[5] Kannaiyan K, Ragunathan L, Sakthivel S, Sasidar AR, Muralidaran. Venkatachalam GK. (2015). Surgical site infections due to rapidly growing Mycobacteria in puducherry India. J Clin Diagn Res.

[6] Rajkumar JS, Vinoth A, Akbar S (2018). Non-tuberculous *Mycobacterium* as a causative factor in port site wound infection-A Case Report. Surg Med Open Acc J.

[7] Rasnake MS, Dooley DP (2006). Culture-negative surgical site infections. Surg Infect (Larchmt).

[8] Leão SC, Viana-Niero C, Matsumoto CK (2010). Epidemic of surgical-site infections by a single clone of rapidly growing *Mycobacteria* in Brazil. Future Microbiol.

[9] Pescitelli L, Galeone M, Tripo L, Prignano F (2015). Cutaneous non-tuberculous mycobacterial infections: clinical clues and treatment options. Curr Treat Options Infect Dis.

[10] Naskar B, Bakshi S, Mandal T (2020). Management of infections with *Mycobacterium* other than tuberculosis as a complication of surgical procedures. Int J Surg.

[11] Shah AK, Gambhir RP, Hazra N, Katoch R (2010). Non-tuberculous mycobacteria in surgical wounds a rising cause of concern?. Indian J Surg.

[12] Kalaiarasan E, Thangavelu K, Krishnapriya K, Muthuraj M, Jose M, Joseph NM (2020). Diagnostic performance of real time PCR and MALDI-TOF in the detection of non-tuberculous mycobacteria from clinical isolates. Tuberculosis.

[13] Huh HJ, Kim SY, Jhun BW, Shin SJ, Koh WJ (2019). Recent advances in molecular diagnostics and understanding mechanisms of drug resistance in non-tuberculous mycobacterial diseases. Infect Genet Evol.

[14] Forbes BA, Hall GS, Miller MB (2018). Practice guidelines for clinical microbiology laboratories: *Mycobacteria*. Clin Microbiol Rev.

[15] Gopalaswamy R, Shanmugam S, Mondal R, Subbian S (2020). Of tuberculosis and non-tuberculous mycobacterial infections–a comparative analysis of epidemiology, diagnosis and treatment. J Biomed Sci.

[16] Považan A, Vukelić A, Savković T, Kurucin T (2012). Use of immunochromatographic assay for rapid identification of *Mycobacterium* tuberculosis complex from liquid culture. Bosn J Basic Med Sci.

[17] Martin A, Bombeeck D, Fissette K (2011). Evaluation of the BD MGIT TBc Identification Test (TBc ID), a rapid chromatographic immunoassay for the detection of *Mycobacterium tuberculosis complex* from liquid culture. J Microbiol Method.

[18] Rodriguez-Temporal D, Rodríguez-Sánchez B, Alcaide F (2020). Evaluation of MALDI biotyper Interpretation criteria for accurate identification of non-tuberculous mycobacteria. J Clin Microbiol.

[19] Alcaide F, Amlerová J, Bou G (2018). How to: identify non-tuberculous *Mycobacterium* species using MALDI-TOF mass spectrometry. Clin Microbiol Infect.

[20] Gaber A, Hamed H, Elsawy E (2017). Comparison between real-time polymerase chain reaction and DNA-microarray in detection and identification of *Mycobacterium* species. Gene Mol Res.

[21] Duarte RS, Lourenço MC, Fonseca Lde S (2009). Epidemic of post-surgical infections caused by *Mycobacterium massiliense*. J Clin Microbiol.

[22] Sharma P, Singh D, Sharma K, Verma S, Mahajan S, Kanga A (2018). Are we neglecting non-tuberculous mycobacteria just as laboratory contaminants? time to reevaluate things. J Pathog.

[23] Veraldi S, Spigariolo CB, Cusini M, Nazzaro G, Gianotti R (2020). Skin infections by *Mycobacterium chelonae* following mesotherapy: a report of two cases and review of the literature. J Cosmet Dermatol.

[24] Machado GE, Matsumoto CK, Chimara E (2014). Multilocus sequence typing scheme versus pulsed-field gel electrophoresis for typing *Mycobacterium abscessus* isolates. J Clin Microbiol.

[25] Hoza AS, Mfinanga SG, Rodloff AC, Moser I, König B (2016). Increased isolation of non-tuberculous mycobacteria among TB suspects in Northeastern, Tanzania: public health and diagnostic implications for control programmes. BMC Res Notes.

[26] Watanabe Pinhata JM, Lemes RA, Simeão FCDS, Souza AR, Chimara E, Ferrazoli L (2018). Use of an immunochromatographic assay for rapid identification of *Mycobacterium tuberculosis* complex clinical isolates in routine diagnosis. J Med Microbiol.

[27] Kandhakumari G, Stephen S (2017). Evaluation of a new rapid kit, BD MGIT TBc identification test for confirmation of *mycobacterium tuberculosis* complex. Indian J Pathol Microbiol.

[28] Buckwalter SP, Olson SL, Connelly BJ (2016). Evaluation of matrix-assisted laser desorption ionization-time of flight mass spectrometry for identification of *mycobacterium* species, nocardia species, and other aerobic actinomycetes. J Clin Microbiol.

[29] Genc GE, Demir M, Yaman G, Kayar B, Koksal F, Satana D (2018). Evaluation of MALDI-TOF MS for identification of non-tuberculous mycobacteria isolated from clinical specimens in mycobacteria growth indicator tube medium. New Microbiol.

[30] Mareković I, Bošnjak Z, Jakopović M, Boras Z, Janković M, Popović-Grle S (2016). Evaluation of matrix-assisted laser desorption/ionization time-of-flight mass spectrometry in identification of non-tuberculous mycobacteria. Chemotherapy.

[31] Mediavilla-Gradolph MC, De Toro-Peinado I, Bermúdez-Ruiz MP (2015). Use of MALDI-TOF MS for identification of non-tuberculous *Mycobacterium* species isolated from clinical specimens. Biomed Res Int.

[32] Lazzeri E, Santoro F, Oggioni MR, Iannelli F, Pozzi G (2012). Novel primer-probe sets for detection and identification of mycobacteria by PCR-microarray assay. J Clin Microbiol.

[33] Haider M, Banerjee P, Jaggi T (2013). Post-operative sinus formation due to *Mycobacterium abscessus:* a case report. Indian J Tuberc.

[34] Brown-Elliott BA, Wallace RJ, Tichindelean C (2011). Five-year outbreak of community- and hospital-acquired *Mycobacterium porcinum* infections related to public water supplies. J Clin Microbiol.

[35] Paul GR, Leber A, Nemastil CJ (2020). Identification of *Mycobacterium porcinum* in patients with cystic fibrosis: pathogen or contaminant?. J Cyst Fibros.

[36] Sam AS, Ninan MM, Ranjani R, Devanga Raghupathi NK, Balaji V, Michael JS (2020). Non-tuberculous mycobacteria clinical and laboratory diagnosis: experiences from a TB endemic country. Future Sci OA.

[37] Biswal M, Singh G, Jain V (2013). First report in the world of *Mycobacterium bacteremicum* causing a cluster of post-laparotomy surgical wound infections. Antimicrob Resist Infect Control.

[38] Chang HY, Tsai WC, Lee TF, Sheng WH (2021). *Mycobacterium gordonae* infection in immunocompromised and immunocompetent hosts: a series of seven cases and literature review. J Formos Med Assoc.

[39] Sumbul B, Doymaz MZ (2020). A Current microbiological picture of *Mycobacterium* isolates from Istanbul. Turkey Pol J Microbiol.

[40] Sarro YD, Kone B, Diarra B (2018). Simultaneous diagnosis of tuberculous and non-tuberculous *mycobacterial* diseases: time for a better patient management. Clin Microbiol Infect Dis.

[41] Olaru ID, Patel H, Kranzer K, Perera N (2018). Turnaround time of whole genome sequencing for *mycobacterial* identification and drug susceptibility testing in routine practice. Clin Microbiol Infect.

[42] Alcolea-Medina A, Fernandez MTC, Montiel N (2019). An improved simple method for the identification of *mycobacteria* by MALDI-TOF MS (matrix-assisted laser desorption- ionization mass spectrometry). Sci Rep.

[43] Fernández-Esgueva M, Fernández-Simon R, Monforte-Cirac ML, López-Calleja AI, Fortuño B, Viñuelas-Bayon J (2021). Use of MALDI-TOF MS (Bruker Daltonics) for identification of *Mycobacterium* species isolated directly from liquid medium. Enferm Infecc Microbiol Clin (Engl Ed).

[44] Huang TS, Lee CC, Tu HZ, Lee SS (2018). Rapid identification of mycobacteria from positive MGIT broths of primary cultures by MALDI-TOF mass spectrometry. PLoS ONE.

[45] Fukushima M, Kakinuma K, Hayashi H, Nagai H, Ito K, Kawaguchi R (2003). Detection and identification of *Mycobacterium* species isolates by DNA microarray. J Clin Microbiol.

[46] Tobler NE, Pfunder M, Herzog K, Frey JE, Altwegg M (2006). Rapid detection and species identification of *Mycobacterium spp* using real-time PCR and DNA-microarray. J Microbiol Methods.

[47] Zimenkov DV, Kulagina EV, Antonova OV (2015). Evaluation of a low-density hydrogel microarray technique for *mycobacterial* species identification. J Clin Microbiol.

[48] Pan’kov SV, Chechetkin VR, Somova OG (2009). Kinetic effects on signal normalization in oligonucleotide microchips with labeled immobilized probes. J Biomol Struct Dyn.

